# Recurrent Hepatocellular Carcinoma in Patient with Crohn's Disease: Incidental or Expected Outcome of Azathioprine?

**DOI:** 10.1155/2015/939136

**Published:** 2015-12-14

**Authors:** Youssef Botros, Mary Mathews, Hiren Patel, Nihar Shah, Walid Baddoura, Andrew de la Torre

**Affiliations:** ^1^Department of Internal Medicine, St. Joseph's Regional Medical Center, New York Medical College, Paterson, NJ 07503, USA; ^2^Department of Gastroenterology, St. Joseph's Regional Medical Center, New York Medical College, Paterson, NJ 07503, USA; ^3^Department of Gastroenterology, Joan C. Edwards School of Medicine, Marshall University, Huntington, WV 25755, USA; ^4^Department of General and Hepatobiliary Surgery, St. Joseph's Regional Medical Center, Paterson, NJ 07503, USA

## Abstract

Hepatocellular carcinoma (HCC) usually occurs in patients with underlying risk factors such as liver cirrhosis and chronic hepatitis B. Although patients with Crohn's disease (CD) are at an increased risk to develop malignancies such as colon cancer, the incidence of HCC in this population is extremely rare. We report a case of 62-year-old male with long history of CD treated with azathioprine (AZA) and aminosalicylic acid (ASA) who was incidentally diagnosed with HCC, for which left hepatectomy was done. Four years later during routine follow-up, patient had another hepatic lesion and underwent resection of the mass. The mechanism of occurrence of HCC in patient with CD is still controversial and may include immune mediated changes and medication related complications. AZA was reported in all case reports of CD that developed HCC. Through this report we hope to explore the complex pathophysiological mechanisms contributing to the development of HCC in the Crohn's disease patient population.

## 1. Introduction

Hepatocellular carcinoma (HCC) is the sixth most common neoplasm worldwide, with about 600,000 deaths annually [1]. HCC usually occurs in patients with underlying risk factor, for example, liver cirrhosis or chronic hepatitis B [2]. Patients with Crohn's disease (CD) are at increased risk of developing cancers, and colon cancer is considered the most important cause of excess mortality among these patients [3]. The incidence of HCC in the Crohn's disease patient population is extremely rare [4]. There are only few cases of HCC in patients with CD reported in literature with only one case reported of recurrent HCC [5]. We report a case of recurrent HCC in a CD patient without underlying liver disease who was treated with azathioprine (AZA) and aminosalicylic acid (ASA).

## 2. Case Presentation

We present a case of a 62-year-old male with 42-year history of small intestinal CD diagnosed at the age of 20, requiring two small bowel resection surgeries during this course of time. Our patient was solely on prednisone therapy for an extended period of time and switched to AZA (150 mg daily) for the last 21 years with relatively well controlled symptoms. During routine follow-up screening tests, he was incidentally found to have left lobe liver mass and was diagnosed with HCC for which he received left lobe hepatectomy at the age of 58. The AZA dose was reduced to 50 mg daily.

On subsequent follow-up, four years after the initial resection, he was found to have liver mass and was admitted to the hospital for further evaluation. On presentation to hospital, the patient appeared well without any evidence of jaundice or stigmata of chronic liver disease. Preoperative blood work including liver profile and viral hepatitis markers was entirely normal: AST 18 U/L (<37 U/L), ALT 16 (<60 U/L), albumin 3.5 g/dL (3.5–5 g/dL), bilirubin 0.8 mg/dL (0.3–1.2 mg/dL), and INR 1.0, alpha-fetoprotein 10.3 ng/mL (<6.1 ng/mL), prothrombin time 13.3 seconds, and platelet count 210/*μ*L.

**Figure 1:**
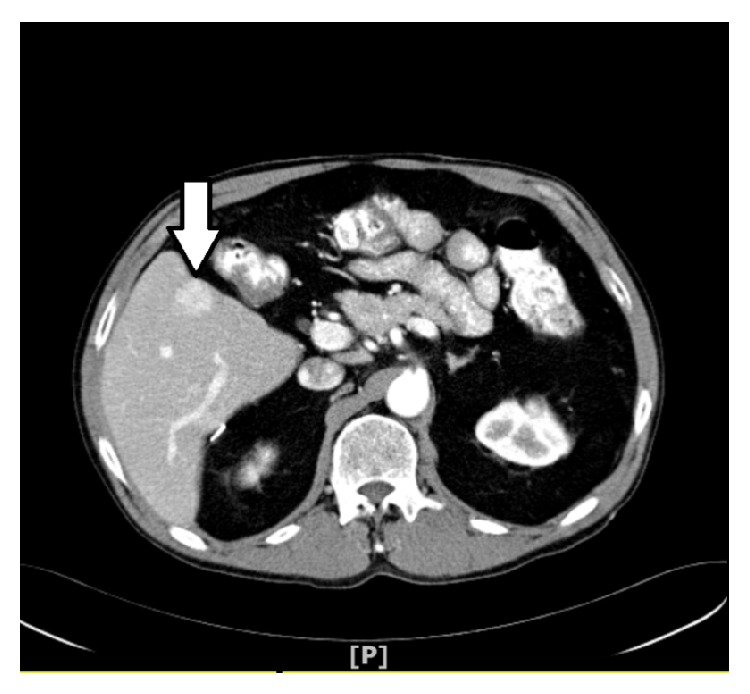
Arterial phase showing 2.2 × 1.6 × 2.1 lesion in segment V.

**Figure 2:**
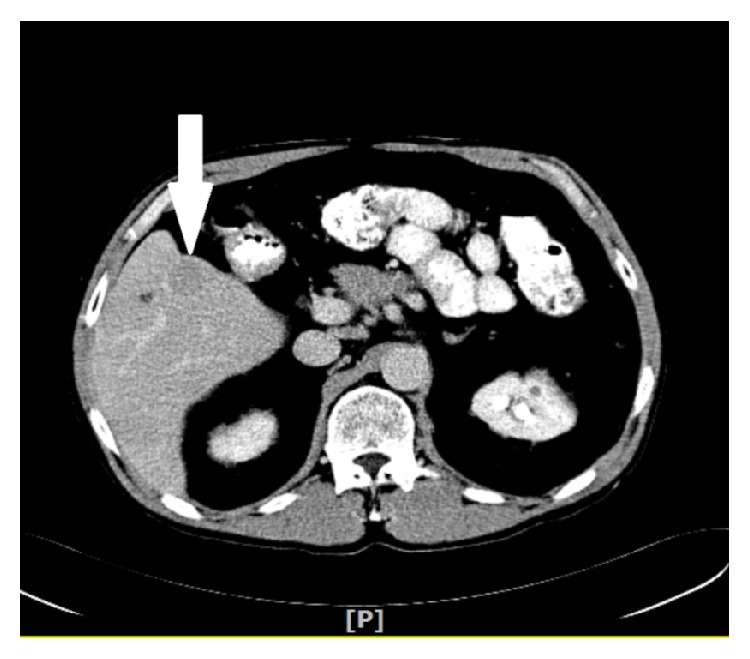
Hepatic lesion showing early wash out in venous phase.

Triple phase contrast enhanced CT of abdomen showed solitary mass in the segment 5 of the right lobe measuring 2.2 × 1.6 × 2.1 cm, with arterial enhancement and early wash out in the venous phase, and no evidence of lymphadenopathy or extrahepatic metastasis (Figures 1 and 2).

**Figure 3:**
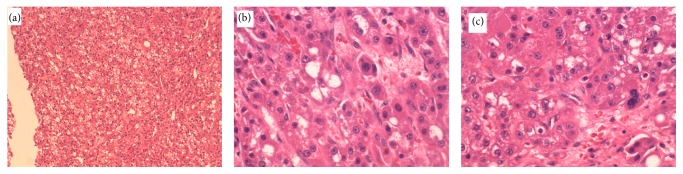
Moderately differentiated HCC, hematoxylin and eosin: low power (a) and high power (b and c).

The decision toward surgical excision of the tumor was made. Patient underwent laparoscopic resection of segment 5 with no postoperative complications. Microscopically, the resected mass showed moderately differentiated hepatocellular carcinoma without evidence of microvascular invasion (Figure 3).

There was no postoperative adverse events and AZA was discontinued and patient was kept on oral budesonide.

## 3. Discussion

The occurrence of HCC among patients with CD is extremely rare, with only about 12 cases reported in the literature [5–16]. Most of the patients with CD who developed HCC received AZA, with a few cases who did not receive AZA but were eventually found to have underlying liver disease [9, 15].

The exact mechanism for the occurrence of HCC in patients with CD without underlying liver disease is not well established. Is it a pathophysiological outcome of Crohn's disease itself? Or is it more related to the treatments of CD? The answers to these scientific queries require further investigations.

AZA use is associated with increased risk of many malignancies including squamous cell skin cancer in patients with rheumatological diseases and lymphoma among IBD patients [17, 18]. However, there are no retrospective studies in medical literature reporting any correlation between the use of AZA and occurrence of HCC in patients with CD.

There have been only two reported cases of hepatocellular carcinoma in patients receiving azathioprine for clinical conditions other than CD, one in patient with ulcerative colitis and the other case in renal transplant patient [19, 20].

The relation between long term use of AZA and development of HCC is still controversial, and the mechanism by which AZA can predispose to HCC is thought to be due to increase in the hepatic cells turnover. Arber et al. demonstrated this effect on animals by injecting rats with AZA and measuring the distance of labeled liver cells from the portal space and found increase in the hepatocytes' and littoral cells' streaming velocities in the AZA treated rats compared to the control group. This can possibly lead to aberrant cell growth via abnormal mechanisms [21]. AZA can also precipitate to cancers via decreasing the immunity against cancer cells and increasing the susceptibility to oncogenic viruses [10].

Other possible mechanisms for the occurrence of HCC in CD patients have been postulated; some endothelial-cell adhesion molecules and chemokines that are restricted to the gut under normal condition become abnormally expressed in the liver in IBD patients. This can lead to immune response mediated by mucosal T cells, against these antigens and subsequently hepatic damage [22].

Another reported pathophysiological mechanism is a correlation between the gut microbiota in patients with CD and occurrence of HCC. Fox et al. showed that gut microbiota can define the risk of HCC development in response to environmental factors including carcinogenic chemicals and hepatitis virus transgenes. Colonization of gut with harmful bacteria is thought to alter bowel mucosal integrity facilitating the passage of harmful bacteria to portal circulation. Also this change in gut microbiota can increase secretion of cytokines acting on the liver and upregulating the transcription of numerous cytokines and receptors that act as key regulator of hepatic injury carcinogenesis [23, 24].

In conclusion, there appears to be complex interplay of pathophysiological mechanisms in patients with CD, leading to the development of HCC while on AZA therapy.

Gastroenterologist should be aware of this rare association between using AZA in CD and development of HCC. We hope that this intriguing area receives robust scientific inquiry in order to establish a strong correlation and find out if patients on AZA for CD should have screening for HCC.
